# A Teenage Girl with Acute Dyspnea and Hypoxemia during Red Blood Cell Transfusion

**DOI:** 10.1155/2016/9372678

**Published:** 2016-11-06

**Authors:** U. Pandee, P. Tanpowpong, P. Thongpo

**Affiliations:** ^1^Department of Pediatrics, Faculty of Medicine Ramathibodi Hospital, Mahidol University, Bangkok, Thailand; ^2^Ramathibodi School of Nursing, Faculty of Medicine Ramathibodi Hospital, Mahidol University, Bangkok, Thailand

## Abstract

Transfusion-related acute lung injury (TRALI) can cause morbidity and mortality. We present the case of teenager who developed dyspnea and hypoxemia few hours after red cell transfusion. After being admitted for close monitoring and oxygen therapy, her symptoms spontaneously resolved. Message: dyspnea during red cell transfusion should raise the suspicion of TRALI.

## 1. Introduction

TRALI is thought to be uncommon but considered as a potential leading cause of transfusion-related morbidity and mortality [1]. However, the condition may likely be underdiagnosed. The incidence of TRALI is estimated to be between 0.08% and 15% in patients receiving blood transfusions [2]. In children, Canadian Blood Service reported TRALI in 6 cases per 100,000 red blood cell transfusions [3]. Nowadays, the US Food and Drug Administration acknowledges this condition as a leading cause of transfusion-related mortality. If it occurred, TRALI has a mortality of 6–9% [4].

## 2. Case Presentation

A 16-year-old female underlying Hodgkin's lymphoma was seen in the emergency department with a complaint of acute dyspnea and oxyhemoglobin desaturation 2 hours after the initiation of leukocyte-poor packed red cell transfusion due to significant anemia. Her vital signs showed temperature of 38°C, pulse rate of 140/minute, respiratory rate of 40/minute, blood pressure 110/70 mmHg, and oxygen saturation of 80% at room air. Her body weight was 67 kilograms and height 162 centimeters. Physical examination showed pale conjunctiva without icteric sclera. Bilateral crackles at both lower lungs were noted without jugular venous distention or gallop rhythm of the heart. No hepatosplenomegaly was noted. Maculopapular rash on both extremities was observed. Her neurological examination was unremarkable.

**Figure 1:**
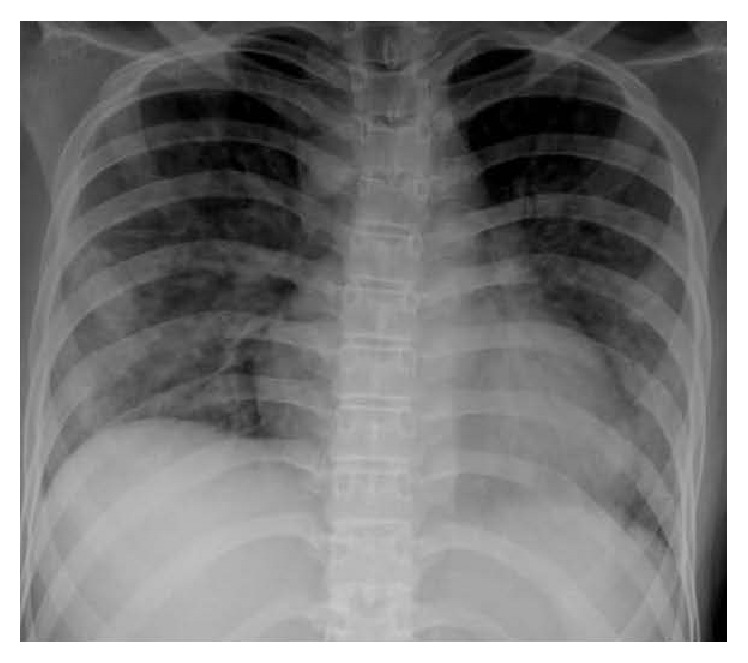


The blood transfusion was then stopped. She received oxygen via mask with reservoir bag with FiO_2_ of 1.0, and the oxygen saturation increased to 98-99%. Intramuscular adrenaline, intravenous chlorpheniramine, and hydrocortisone were given because a diagnosis of anaphylaxis could not be completely ruled out. Arterial blood gas revealed severe hypoxemia (PaO_2_ 37 mmHg, PaO_2_/FiO_2_ ratio = 37) and oxygen saturation 70% with normal PaCO_2_ and bicarbonate. Complete blood count showed hemoglobin 6 g/dL, hematocrit 18.2%, white blood cell count 2.7 × 10^9^/L (neutrophils 72% and absolute neutrophil count (ANC) 1.9 × 10^9^/L), lymphocytes 12%, monocytes 14% (absolute monocyte count 0.38 × 10^9^/L), band form (2%), and platelet count 17 × 10^9^/L. There were no signs or symptoms of hemolysis. Serum electrolytes and glucose were normal. Due to an initial concern of anaphylaxis, serum tryptase was sent and later reportedly normal. Chest radiography demonstrated bilateral pulmonary infiltration (Figure 1). A differential diagnosis of transfusion-related complications, both transfusion associated circulatory overload (TACO) and transfusion-related acute lung Injury (TRALI), were raised, but there was no sign of circulatory overload, by bedside assessment (her body weight was repeated to 67.1 kilograms, intake/output was 226/180 mL, no engorgement of neck vein, and no hepatomegaly); hence a diagnosis of TACO was unlikely. The patient was admitted to the general pediatric ward with a suspicion of TRALI.

Oxygen supplementation and supportive treatment were continued. She clinically improved 36 hours after admission with close monitoring; oxygen therapy was ceased. No diuretics, medications, or additional blood transfusions were required during hospitalization. Follow-up chest radiography showed significant improvement. She was discharged home the next day. Four weeks after the admission, she remained asymptomatic without any further reported symptoms.

## 3. Discussion

TRALI is defined as a sudden onset of respiratory distress during or after the blood transfusion. The diagnosis predominantly consisted of acute respiratory distress, dyspnea, hypoxemia, and pulmonary edema with bilateral pulmonary infiltration on chest radiography.

Most reviews accept that TRALI is a clinical diagnosis; therefore no laboratory studies are required [5]. The definition of TRALI is derived from acute lung injury (ALI) provided by the Canadian Consensus Group [6]. TRALI can be defined as the following:

1. ALI is defined as
  acute onset,
  hypoxemia (PaO_2_/FiO_2_ < 300 or oxygen saturation < 90% on room air or other clinical evidence of hypoxemia),
  bilateral pulmonary infiltration on chest radiography,
  no evidence of circulatory overload.
2. No existing ALI before blood transfusion.
3. Occurring during or within 6 hours of blood transfusion.
4. No temporal relationship to alternative risk factors for ALI which include aspiration, pneumonia, toxic inhalation, lung contusion, near drowning, severe sepsis, shock, multiple trauma, burn injury, acute pancreatitis, cardiopulmonary bypass, or drug overdose.

Our patient developed acute respiratory symptoms two hours after the initiation of red blood cell transfusion with severe hypoxemia and bilateral lung infiltration. She had no preexisting ALI before the transfusion and showed no clinical signs of circulatory overload. Furthermore, obvious alternative risk factors were not found prior to the ALI episode. Therefore, a clinical diagnosis of TRALI was eventually made.

Two pathophysiologic mechanisms have been proposed for TRALI. The first is named the “antibody hypothesis”; the pathogenesis relates to an infusion of donor antibodies that is recognized by leukocyte antigens in the transfused host [2]. Antibodies toward human leukocyte antigen (HLA) or human neutrophil antigen (HNA) have been implicated. The second hypothesis is called the “two-hit or two-event” model [7] which proposed that recipient's neutrophils are primed by a preexisting clinical condition. This model states that the first event is due to the clinical condition of the patient which causes activation of the pulmonary endothelium resulting in firm adhesion of neutrophils. Adherent neutrophils are primed and hyperreactive, primed (adherent) neutrophils. The second event, a priming agent, then activates these neutrophils. The hard part is that priming agents activate primed (adherent) neutrophils; the nomenclature is confusing. Furthermore, the two-event model also includes antibodies to HLA (both class I and class II) and HNA as the second event which encompasses all known models of TRALI. The final common pathway of both mechanisms is increased pulmonary capillary permeability, which results in movement of plasma into the alveolar space causing pulmonary edema.

Differential diagnosis should include other causes of pulmonary edema. Anaphylaxis or septic transfusion reaction can also present with a relatively similar clinical presentation to TRALI [8]. Chest radiography will aid in the differential diagnosis. Distinguishing between hydrostatic pulmonary edema caused by TACO and pulmonary infiltrations caused by TRALI is a challenge. Clinical signs of volume overload and specific investigations such as echocardiogram or brain natriuretic peptide have been used to help in the diagnosis [9]. TACO usually presented with volume overload in conjunction with significant pulmonary infiltrations or edema. Our patient did not have invasive cardiovascular monitoring; however by bedside clinical assessment did not show sign of circulatory overload: stable body weight, balance intake/output, no engorgement of neck vein, and no hepatomegaly; hence TACO was unlikely.

Supportive treatment is a foundation in managing patients with TRALI which includes oxygen supplementation, noninvasive ventilation, mechanical ventilation, and hemodynamic supports. Patients with TRALI are expected to improve within 48–96 hours, and the chest radiograph abnormalities should recover in 4–7 days [4]. Our patient improved in 36 hours with oxygen support and did not require mechanical ventilation or hemodynamic support which may be due to an early detection. Preventive strategy of TRALI is to reduce the risk of exposure by excluding “at risk” donors and pooling of high plasma volume products [10].

## 4. Conclusion

We reported a teenage girl with Hodgkin's lymphoma who developed acute respiratory distress, low grade fever, rash, hypoxemia, and bilateral pulmonary infiltration after few hours of red blood cell transfusion. Transfusion-related complications such as anaphylaxis, septic transfusion reaction, TACO, and TRALI are conditions to be considered. This patient likely suffered from TRALI by established clinical diagnostic criteria. If suspected or diagnosed patients are with TRALI, respiratory and circulatory supports with close monitoring are crucial because this condition can lead to significant morbidity and mortality.

## Abbreviations

ALI: Acute lung injury
TRALI: Transfusion-related acute lung injury
TACO: Transfusion associated circulatory overload.
